# Food insecurity and its determinants among adults in North and South India

**DOI:** 10.1186/s12937-022-00831-8

**Published:** 2023-01-09

**Authors:** Anjali Ganpule, Kerry Ann Brown, Manisha Dubey, Nikhil Srinivasapura Venkateshmurthy, Prashant Jarhyan, Avinav Prasad Maddury, Rajesh Khatkar, Himanshi Pandey, Dorairaj Prabhakaran, Sailesh Mohan

**Affiliations:** 1 Centre for Chronic Disease Control, New Delhi, India; 2 University of Exeter, Exeter, UK; 3 Public Health Foundation of India, New Delhi, India; 4 London School of Hygiene and Tropical Medicine, London, UK; 5 Deakin University, Melbourne, Australia

**Keywords:** Food insecurity, Indian adults, Dietary diversity, BMI

## Abstract

**Background:**

Food insecurity is a major public health problem worldwide. In India, there are limited food insecurity assessment studies using a conventionally accepted method like the Food Insecurity Experience Scale (FIES), developed by the Food and Agricultural Organization (FAO). This study aims to measure food insecurity using the FIES and explore its determinants and association with body mass index (BMI) among Indian adults.

**Methods:**

In a cross-sectional study, we used FIES to measure food security in a sample of 9005 adults residing in North and South India. Using questionnaires, socio-demographic factors, dietary intake and food security data were collected. The dietary diversity scores (FAO-IDDS) and food insecurity scores (FAO-FIES) were calculated. Body size was measured and BMI was calculated.

**Results:**

The mean age of the study participants was 52.4 years (± 11.7); half were women and half resided in rural areas. Around 10% of the participants reported having experienced (mild or moderate or severe) food insecurity between October 2018 and February 2019. Dietary diversity (measured by FAO’s Individual Dietary Diversity Scores, IDDS) was low and half of the participants consumed ≤ 3 food groups/day. The mean BMI was 24.7 kg/m^2^. In the multivariate analysis, a lower IDDS and BMI were associated with a higher FIES. The place of residence, gender and wealth index were important determinants of FIES, with those residing in South India, women and those belonging to the poorest wealth index reporting higher food insecurity.

**Conclusion:**

Food security is understudied in India. Our study adds important evidence to the literature. Despite having marginal food insecurity, high prevalence of low diet quality, especially among women, is disconcerting. Similar studies at the national level are warranted to determine the food insecurity situation comprehensively in India and plan appropriate policy actions to address it effectively, to attain the key Sustainable Development Goals (SDG).

**Supplementary Information:**

The online version contains supplementary material available at 10.1186/s12937-022-00831-8.

## Introduction

Food security entails access to sufficient, safe and nutritious food that meets people’s dietary needs and food preferences, for leading an active and healthy life [1]. Despite India being among the fastest growing economies in the world and ranking second worldwide in farm output [2], the country still faces hunger and diet quality-related issues. Not surprisingly, India is ranked 101 out of 116 countries in the most recent Global Hunger Index report [3]. It shows that India is still lagging behind when it comes to meeting hunger-related United Nations Global Sustainable Development Goals (e.g., zero hunger (Goal 2), good health and well-being (Goal 3) and in supply of sufficient quantities of food to ensure adequate availability [4]. An examination of the food insecurity dynamics based on the National Sample Survey data on household consumer expenditure in India since year 2000 revealed that the overall rate of food insecurity has declined, but at a very slow pace [5]. Thus, monitoring the food insecurity situation and taking immediate policy actions is a public health priority for India.

In the past, multiple proxy measures like anthropometry [6], wealth index and literacy [7] have been used to assess food insecurity, mainly as food adequacy. However, food insecurity in the Indian context requires measurement of both food inadequacy [4] and micro-nutrient deficiency, considering that both are highly prevalent [8]. Studies around the world also demonstrate the need for this as food insecurity is closely linked to the quality of diets and malnutrition in all its forms [9, 10]. Thus, measuring food insecurity using the Food Insecurity Experience Scale (FIES) [11], which captures both hunger and micronutrient deficiencies, is appropriate in the Indian context. The FAO developed the FIES tool in 2016 [11], which is globally accepted as a robust and cost-effective indicator or measure of food insecurity [11, 12]. It allows the measurement of mild, moderate and severe food insecurity. Mild food insecurity is experienced when hunger is addressed through the intake of cereal-based foods but there is a lack of dietary diversity and variety of food, while severe food insecurity is experienced when people are hungry as they do not get enough food to eat. Validation studies of FIES in India were conducted in 2012 and 2014 among 3,000 individuals and published in the State of Food Security and Nutrition in the World report (SOFI) [13]. However, very few studies have used FIES to assess food insecurity in India [14]. Understanding the country-specific context, drivers, and determinants of food insecurity is important [15]. This can support the planning of targeted interventions as well as effective policies and programs.

Multiple studies show an association of under-and overnutrition with food insecurity [16, 17]. Thus, there is a need to study its association with a nutritional outcome like BMI through country-specific studies [18], particularly in low-middle income countries (LMICs) [19]. The current study addressed these gaps in the evidence base by measuring the prevalence of food insecurity using FIES, studying its association with socio-demographic factors, economic factors, dietary diversity and BMI among residents of rural and urban households in north and south India.

## Materials and methods

### Study design

**Fig. 1:**
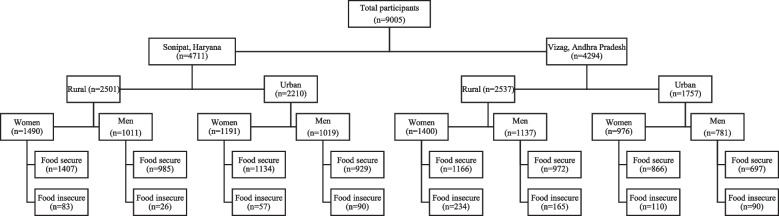
Flowchart of the study participants. Note: For the present study, cross-sectional data from a larger longitudinal study UDAY are presented. A flow diagram of participants in this longitudinal study has been published elsewhere (Mohan, Set al. 2018). There was no exclusion of the participants. All 9005 participants responded and were included in the study. Socio-demographic, dietary and food security data are available for all the participants based on which the results are presented. BMI data was available for 8718 participants

The analysis presented in this paper is based on data from the UDAY cohort study’s baseline follow-up survey, conducted during October 2018-February 2019, among adult members of urban and rural households in Sonipat (north India) and Vizag (south India) (Fig. 1). The methodology of the surveys in the UDAY cohort has been published previously [20]. Briefly, the study enrolled 12,000 individuals aged ≥ 30 years and was established to improve the prevention, detection and management of diabetes and hypertension.

### Measurements

Trained research staff carried out the measurements, which were closely monitored for quality assurance.

### Demographics

Information on age, sex, residence (urban or rural), state (Haryana or Andhra Pradesh), household assets were collected through a questionnaire.

### Food insecurity

Food insecurity was measured using the FAO’s FIES eight-item scale, which asks participants to self-report food-related behaviours and experiences associated with increasing difficulties in accessing food due to resource constraints [11]. As per the standard protocol, participants who responded with a “yes” to 1) being worried about not having enough food or 2) were unable to eat healthy and nutritious food or 3) eating only a few kinds of food, were scored as having mild food insecurity. Those who responded with a “yes” to 4) to skip a meal or 5) ate less or 6) ran out of food, were scored as having moderate food insecurity, while those who responded with a “yes” to 7) were hungry but did not eat or 8) went without eating for a whole day, were scored as having severe food insecurity. The validity of FIES scores as a continuous variable was checked using the Rasch model, as suggested by the FAO (2016). Infit for all questions was within the limit (< 1.3), as recommended by FAO, except for an item (Whole day without eating. Infit:1.35). The outfit was within the limit (< 2.0) for all items except for one item (Whole day without eating. Outfit: 3.54). We dropped this item for further analysis as recommended. For the question “You went without eating for a whole day?” there were only 102/9005 responses and the Rasch model infit was > 1.3. Thus, as per the FAO protocol, after removing these responses, we calculated the proportion of participants experiencing total FIES scores (ranging from 1 to 7), which was used as a continuous variable for the multiple regression analysis.

### Dietary diversity

We conducted an individual food consumption survey using the food frequency questionnaire. Using these data, the individual dietary diversity score (IDDS) was calculated to assess the quality of diet [21]. Foods were grouped according to the characteristics and nutrient profile predetermined by the FAO for the IDDS as 1) All starchy staples 2) Legumes 3) Milk and milk products 4) Meat and fish 5) Eggs 6) Dark green leafy vegetables, and 7) Other fruits and vegetables. For a food group to be counted in the dietary diversity analysis, the minimum average quantity was set at ≥ 15 g/d. The maximum score of the IDDS was 7 instead of 9 as we did not separately recall for two groups: vitamin A-rich fruits vegetables and organ meats.

### Body Mass Index (BMI)

Body weight was measured to the nearest 0.1 kg and height to the nearest 0.1 cm, following the standard procedure [22] and BMI was calculated as weight in kilograms divided by height in meters squared.

### Wealth index

Wealth index was calculated separately for participants from rural and urban areas [23] using principal component analysis (PCA), which was based on the ownership of 12 household assets (radio, TV, computer, phone, fridge, bike, scooter, car, washing machine, sewing machine, house, and land), and 5 key housing characteristics (water supply, type of toilet and whether it is shared, cooking fuel, housing material, and source of lighting). The first component in the PCA was extracted and divided into quintiles- the first quintile being the poorest and the fifth being the richest.

### Statistical analysis

Continuous variables are presented as means (standard deviation [SD]) and categorical variables as frequencies (%). Two sample t-test or Mann Whitney U tests were used based on the distribution of the data for examining the differences in the FIES by wealth index, IDDS and BMI. Multivariate linear regression analysis adjusted for age, sex, and place of residence (rural/urban) was done to study the association of various factors such as IDDS, BMI and wealth index with FIES. We performed mediation analysis using the Monte Carlo simulation (MCS) test to estimate the effect of IDDS on FIES through BMI. The statistical analysis was done using Stata version 16.1 (Stata Corp).

The STROBE-Nut checklist is provided as an additional file.

## Results

### Demographics

The study included 9005 participants with a mean age of 52.4 (± SD 11.7) years (Table 1). Around half the participants were women and resided in rural areas. Participants from Sonipat were richer compared to those residing in Vizag, as indicated by the wealth index.

**Table 1:** Sample characteristics and indices of food security

Sample characteristics (n)		Sonipat				Vizag			
		Rural (2501)		Urban (2210)		Rural (2537)		Urban (1757)	
		Women	Men	Women	Men	Women	Men	Women	Men
Age in years	Mean (SD)	52.4 (12.1)	54.7 (12.0)	51.8 (11.8)	53.5 (11.8)	50.5 (10.7)	54.3(11.5)	49.8 (11.9)	53.5(12.4)
Wealth index % (95% CI)	1 (Poorest)	6.7 (5.5–8.1)	6.9 (5.5–8.7)	18.4 (16.3–20.7)	19.0 (16.7–21.6)	33.6 (31.1–36.1)	30.7 (28.1–33.4)	19.8 (17.4–22.4)	18.3 (15.7–21.2)
	2	10.6 (9.1–12.3)	10.4 (8.6–12.4)	11.5(9.8–13.4)	10.8 (9.0–12.9)	29.8 (27.4–32.2)	28.7 (26.1–31.4)	30.6 (27.8–33.6)	28.7 (25.6–32.0)
	3	18.9 (17.0–20.9)	18.0 (15.7–20.5)	16.0 (14.0–18.1)	16.3 (14.1–18.7)	21.6 (19.6–23.9)	22.8 (20.4–25.3)	28.1 (25.3–31.0)	29.1 (26.0–32.4)
	4	28.1 (25.8–30.4)	27.5 (24.8–30.3)	23.0 (20.7–25.5)	22.2 (19.7–24.8)	11.8 (10.2–13.6)	13.5 (11.7–15.7)	15.3 (13.1–17.7)	16.5 (14.1–19.3)
	5 (Richest)	35.8 (33.4–38.2)	37.2 (34.3–40.2)	31.2 (28.6–33.8)	31.7 (28.9–34.6)	3.2 (2.4–4.3)	4.3 (3.3–5.7)	6.3 (4.9–8.0)	7.4 (5.8–9.5)
Dietary diversity (IDDS)	Mean (SD)	3.4 (0.7)	3.4 (0.7)	3.4 (0.8)	3.3 (0.8)	3.4 (0.7)	3.4 (0.7)	4.0 (0.9)	3.9 (0.9)
BMI kg/m^2^	Mean (SD)	25.6 (5.0)	23.5 (4.4)	27.5 (5.3)	25.3 (5.2)	22.7 (4.4)	22.0 (3.8)	27.1 (4.9)	25.2 (4.1)
Food insecurity	FIES > 0 n (%)	83(5.6)	26(2.7)	57(4.8)	90(8.8)	234(16.7)	175(11.8)	110(11.3)	84(8.6)

### Prevalence of food insecurity

**Fig. 2:**
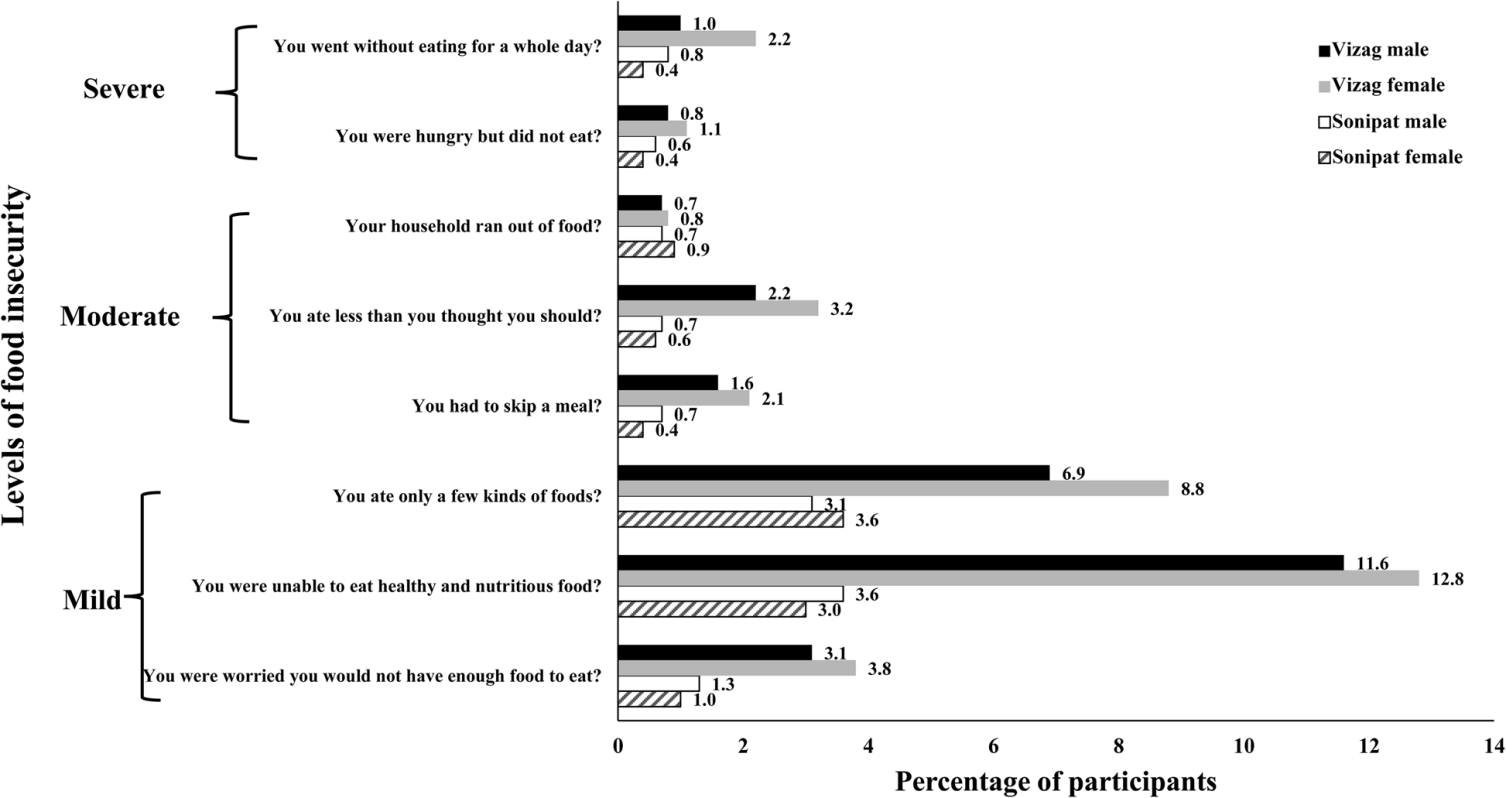
Distribution of the study participants by levels of food insecurity. Legend: The figure describes distribution of participants by the levels of food security as mild, moderate and severe. It shows the food insecurity among men and women in Sonipat and Vizag

About 10% of the participants experienced food insecurity. A higher proportion of participants reported mild FIES than moderate or severe FIES (Fig. 2). Women were more likely to report food insecurity than men (Table 1). Rural residents had higher food insecurity compared to their urban counterparts. Participants from Vizag reported significantly higher food insecurity compared to those in Sonipat.

### Dietary diversity

**Fig. 3:**
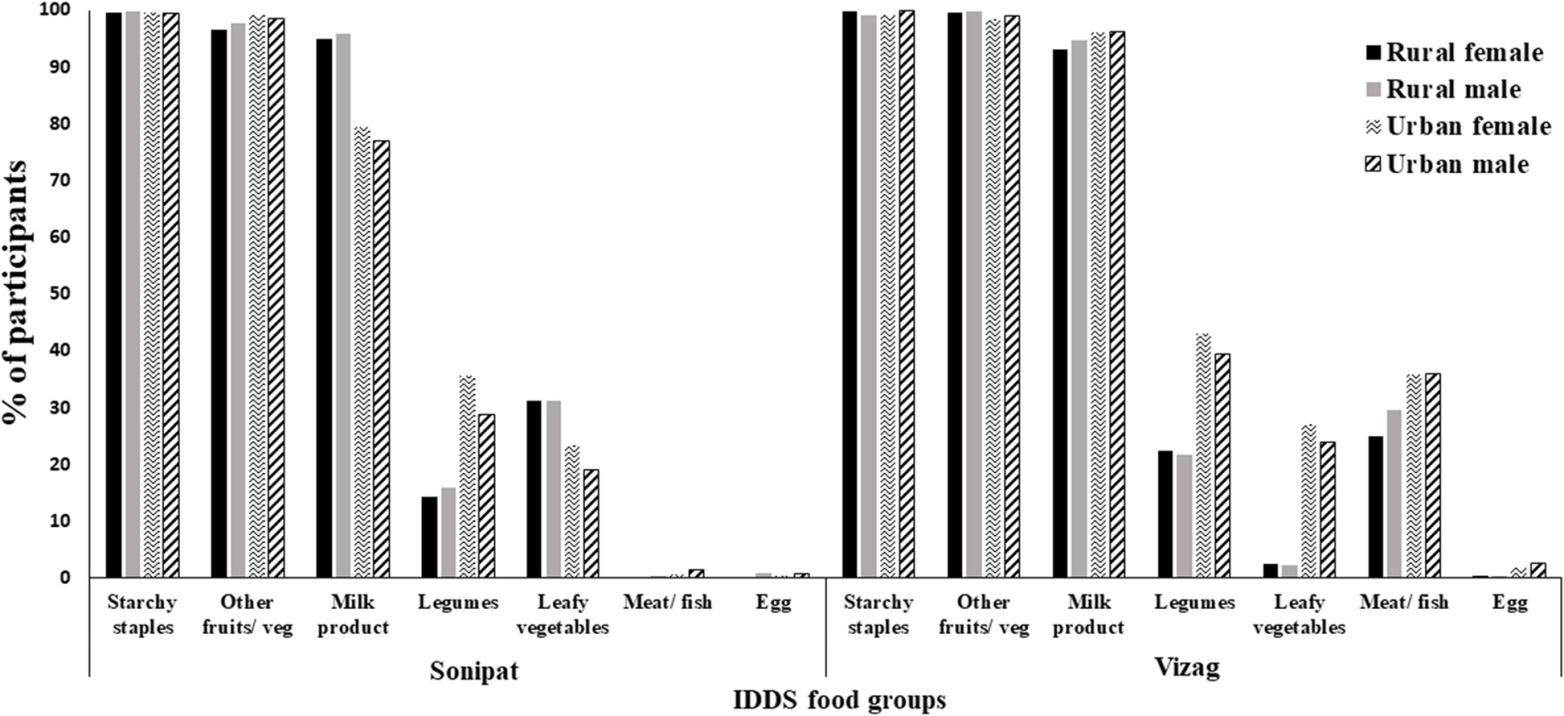
Distribution of the study participants by IDDS food groups. Legend: The figure reports the proportion of participants consuming various dietary diversity food groups in Sonipat and Vizag by gender and place of residence

Dietary diversity was low (mean 3.5 ± SD 0.8). Overall dietary diversity was higher (*p* < 0.05) in Vizag (3.7 ± SD 0.9) compared to Sonipat (3.3 ± 0.8). Dietary diversity was lower in rural compared to urban areas (Table 1). About 90% of participants from Sonipat consumed vegetarian diets, while in Vizag > 80% consumed nonvegetarian diets consisting of eggs and fish, while meat was consumed less frequently. The IDDS food groups consumed daily were calorie-rich food groups, i.e., starchy staples, other fruits and vegetables. Protein-rich foods such as dairy were consumed daily (Fig. 3). Consumption of nutrient-rich food groups, i.e., green leafy vegetables, vitamin A and C-rich fruits and vegetables and non-vegetarian foods was low. A higher proportion of women consumed green leafy vegetables and legumes than men. In Vizag, men consumed nonvegetarian foods in higher proportions than women.

### Body mass index (BMI)

The mean BMI was 24.8 ± SD 5.7 kg/m^2^ (*p* < 0.001 for all differences). Mean BMI was higher in women, participants from urban areas and in Sonipat (*p*-value < 0.001) (Table 1).

### Mediation and regression analysis

**Fig. 4:**
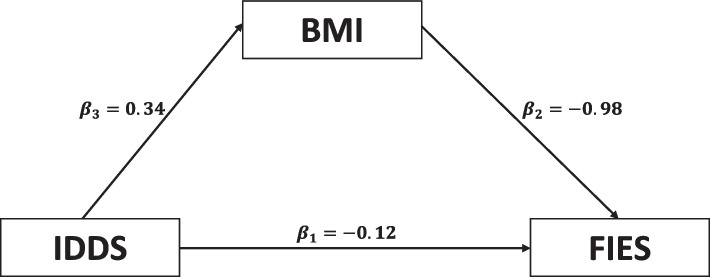
Mediation analysis between dietary diversity, body mass index and food insecurity scores. Legend: The figure shows the results of mediation analysis between BMI, IDDS and FIES. *p*-value for B1 is 0.113 (insignificant) and for B2 and B3, it is < 0.001 (significant). This indicates that IDDS is associated with food insecurity through BMI

We studied the association between IDDS, BMI and FIES using mediation analysis. BMI and IDDS were directly and significantly associated with each other, while both were inversely associated with FIES. Thus, those who had higher IDDS and BMI reported less food insecurity. The indirect effect of IDDS on FIES (via BMI) was found to be around 35% (*p* < 0.01), indicating that the association between IDDS and FIES was indirectly mediated through BMI (Fig. 4). In multivariate linear regression analysis (adjusted for state, residence, age, sex and wealth index), IDDS and BMI were inversely associated with FIES. The age of the participants and urban/rural residence were not significant in the multivariate model, while the state of residence, sex, and wealth index were important determinants of FIES. Women reported experiencing significantly higher food insecurity than men. Further, those residing in Vizag and those belonging to the poorest wealth index had higher food insecurity (Table 2).

**Table 2:** Association of different factors with FIES in the multivariate analysis

FIES Rasch	Beta coefficients	95% CI	*p*-value
Dietary diversity	-0.035	-0.054–0.016	< 0.001
Body mass index	-0.009	-0.013–0.006	< 0.001
Sex			
Women			
Men	-0.047	-0.078–0.016	0.003
Age	0.001	-0.000–0.002	0.061
Wealth index			
Poorest			
Poor	-0.256	-0.304–0.208	< 0.001
Middle	-0.337	-0.384–0.289	< 0.001
Rich	-0.394	-0.443–0.344	< 0.001
Richest	-0.398	-0.449–0.345	< 0.001
State			
Andhra Pradesh			
Haryana	-0.147	-0.19–0.103	< 0.001
Residence			
Urban			
Rural	0.020	-0.012–0.052	0.231

## Discussion

The overall prevalence of food insecurity was low at about 10%. A relatively lower proportion (3%) of the participants reported moderate or severe food insecurity, while mild food insecurity was the highest, being reported by 6.4% of the participants. Dietary diversity was low with lesser consumption of nutrient-rich food groups like vegetables, fruits and protein-rich foods. Most of the participants met their daily calorific requirements through the consumption of starchy staples and starchy vegetables. The mean BMI was 24.7 kg/m^2^, which was directly associated with diet diversity scores. Both diet diversity and BMI were inversely associated with FIES. Further, we found gender and economic status to be significant determinants of FIES among the participants.

Compared to the levels reported in the SOFI report (2020), which shows a high prevalence (24%) of severe food insecurity, the prevalence of moderate or severe food insecurity was low in our study population. One likely reason for this difference could be that the participants in this study resided in economically stable states. Haryana ranks 12^th^, while Andhra Pradesh ranks 27^th^ among 36 Indian states in the Human Development Index (HDI) of the Government of India 2021 [24]. Our finding of mild food insecurity even in economically stable states is disconcerting. Levels of food insecurity may be much higher in less economically stable states of India. It is thus necessary to establish a baseline and monitor the levels of food insecurity at regular intervals through periodic surveys in all states of India. This is required to plan rigorous and continuous remedial measures to address food insecurity effectively. Present-day threats like COVID-19 pandemic, which results in both health and economic downturns and shocks like climate change induced global warming, that affect all aspects of the food systems, underline the need for such a strategy, as populations can rapidly move between states of being food insecure or food secure.

We found that at the individual level, FIES appropriately measured both hunger and micronutrient deficiencies, and thus is applicable to the Indian context. The tool is globally accepted and recommended for monitoring achievements related the SDG goals [25]. It applies to both developed and developing countries as it is pre-tested and validated using data from 147 countries [26]. The findings of mild food insecurity also likely indicate limited access and availability of diverse healthy and nutritious foods. The association of FIES with economic status additionally hints at the affordability issues. Earlier studies have also reported that affordability and accessibility of healthy foods [27, 28] can affect food insecurity.

There are efforts at the national level being undertaken to address these issues. For example, the Government of India has undertaken many reforms of the country’s social safety net programs to improve delivery on nutrition and food security targets [29]. The EAT right campaign of the Food Safety and Standards Authority of India (FSSAI 2021), has brought sustainability into the national nutrition agenda. Additionally, studies suggest the need to expand the food subsidy programs under the National Food Security Act (NFSA) [30], and the need to include the nutrient-rich food groups in these programs [31]. To improve consumer practices and awareness related to fruit and vegetable consumption, specific interventions [32] and nutrition education campaigns [33, 34] have also been found to be effective to a certain extent. Overall, a comprehensive holistic approach with targeted interventions will be helpful for improving the consumption of nutrient-rich foods and attaining food security over time.

In the past, studies have reported inconsistent associations of food insecurity with undernutrition and overnutrition. For example, a meta-analysis from 12 countries reported that food insecurity increases the risk of underweight and stunting in children and adolescents [16]. A longitudinal mixed-method study among adults in the United States reported that food insecurity was associated with an increase in BMI [35]. In a review of 13 studies, which explored the relationship between food insecurity and overweight/obesity in LMICs, four found a positive association between food insecurity and obesity/overweight; five found no association; and the remaining study found a negative association [19]. Our study showed that those who had higher food insecurity had higher BMI. This was irrespective of the socio-demographic and economic factors.

One of the key findings is the effect of gender on the food insecurity experience. Women reported higher food insecurity than men. A systematic review and meta-analysis of gender differences in food security revealed that women-headed households reported higher food insecurity [36]. Even though women contribute to one-half of the world’s food production, they face many inequities, such as access to a lower amount of food and a lower proportion of nutrient-rich food. A few studies have reported gender differences in food and calorie allocation at the household level [37, 38]. We found that women, especially from rural area, had lower consumption of nutrient-rich foods such as dairy, fruits-vegetables and nonvegetarian foods. These findings warrant gender-sensitive policies to ensure that all have equal access to nutrient-rich diets.

At present, food systems are facing challenges due to disruptions induced by the COVID-19 pandemic [39], which has resulted in decreased economic activity, widespread unemployment, and widening health inequalities [40]. To address such shocks that disrupt food systems, effective policies are necessary both at the local and global levels [41]. To achieve the Sustainable Development Goal of Zero Hunger by 2030 and to tackle food insecurity, a more responsive food system that meets people’s needs is warranted. This should be aligned with contextually relevant research and targeted policy efforts to make the food system more climate-resilient, nutrition-sensitive and sustainable [42]. Further, the Global Panel on Agriculture and Food Systems for Nutrition [43] suggests enhancing and repurposing food-based dietary guidelines and new measures of successes to guide policy decisions, and a new set of incentives to rebalance food prices, to simultaneously address challenges of affordability, availability, consumer demand, and sustainability, which have a direct and significant impact on food security. 

## Conclusion

Our study reports mild food insecurity in adults from relatively well-developed states in India. It underlines the need for regular monitoring of the food insecurity situation in India along with the measurement of diet quality and malnutrition, using robust methods. Policies to reduce gender inequalities and increase public awareness about healthy and nutritious diets are warranted. 

## Supplementary Information

**Additional file 1.** STROBE checklist.

**Additional file 2:Supplementary Table 1.** Rasch infit and outfit for FIES scale.

## Data Availability

The datasets used and/or analysed during the current study are available from the corresponding author on reasonable request.

## Abbreviations

BMI: Body Mass Index
FAO: Food and Agricultural Organization
FIES: Food Insecurity Experience Scale
IDDS: Individual Dietary Diversity Scores
