# Supplementary material for: Food insecurity and its determinants among adults in North and South India
Source: Nutr J. 2023 Jan 9;22:2. doi: 10.1186/s12937-022-00831-8 (PMC9827013; doi:10.1186/s12937-022-00831-8)
Supplement: Supplementary file 2 — Additional file 2:Supplementary Table 1. Rasch infit and outfit for FIES scale. [file 12937_2022_831_MOESM2_ESM.docx]

**Supplementary Table 1: Rasch infit and outfit for FIES scale**

| **FIES questions** | **Severity** | **S.E.** | **Infit** | **S.E. Infit** | **Outfit** |
| --- | --- | --- | --- | --- | --- |
| 1. You were worried you would not have enough food to eat? | -0.192 | 0.095 | 0.837 | 0.053 | 0.746 |
| 1. You were unable to eat healthy and nutritious food? | -2.589 | 0.083 | 0.97 | 0.037 | 1.075 |
| 1. You ate only a few kinds of foods? | -1.808 | 0.079 | 0.852 | 0.037 | 0.918 |
| 1. You had to skip a meal? | 0.734 | 0.123 | 0.957 | 0.084 | 0.719 |
| 1. You ate less than you thought you should? | 0.238 | 0.106 | 0.904 | 0.065 | 0.849 |
| 1. Your household ran out of food? | 1.332 | 0.151 | 0.887 | 0.115 | 1.18 |
| 1. You were hungry but did not eat? | 1.441 | 0.157 | 0.983 | 0.121 | 1.572 |
| 1. You went without eating for a whole day? | 0.846 | 0.127 | 1.352 | 0.089 | 3.536 |
